# Notoginsenoside R1 inhibits vascular smooth muscle cell proliferation, migration and neointimal hyperplasia through PI3K/Akt signaling

**DOI:** 10.1038/s41598-018-25874-y

**Published:** 2018-05-15

**Authors:** Haihong Fang, Shilin Yang, Yingying Luo, Cheng Zhang, Yi Rao, Renjing Liu, Yulin Feng, Jun Yu

**Affiliations:** 1 Center for translational Medicine, Jiangxi University of Traditional Chinese Medicine, Nanchang, Jiangxi 330002 China; 2 Department of Physiology and Center for Metabolic Disease Research, Lewis Katz School of Medicine, Temple University, Philadelphia, PA19140 USA; 3 School of Pharmacy, Jiangxi Science and Technology Normal University, Nanchang, Jiangxi 330013 China; 4 Agnes Ginges Laboratory for Diseases of the Aorta, Centenary Institute, University of Sydney, Sydney, Australia; 5 Sydney Medical School, University of Sydney, Sydney, Australia

## Abstract

Restenosis caused by neointimal hyperplasia significantly decreases long-term efficacy of percutaneous transluminal angioplasty (PTA), stenting, and by-pass surgery for managing coronary and peripheral arterial diseases. A major cause of pathological neointima formation is abnormal vascular smooth muscle cell (VSMC) proliferation and migration. Notoginsenoside R1 (NGR1) is a novel saponin that is derived from *Panax notoginseng* and has reported cardioprotective, neuroprotective and anti-inflammatory effects. However, its role in modulating VSMC neointima formation remains unexplored. Herein, we report that NGR1 inhibits serum-induced VSMC proliferation and migration by regulating VSMC actin cytoskeleton dynamics. Using a mouse femoral artery endothelium denudation model, we further demonstrate that systemic administration of NGR1 had a potent therapeutic effect in mice, significantly reducing neointimal hyperplasia following acute vessel injury. Mechanistically, we show that NGR1’s mode of action is through inhibiting the activation of phosphatidylinositol 3-kinase (PI3K)/Akt signaling. Taken together, this study identified NGR1 as a potential therapeutic agent for combating restenosis after PTA in cardiovascular diseases.

## Introduction

Restenosis as a result of intimal hyperplasia is a major cause of coronary artery and peripheral arterial disease^1–4^. Although drug-eluting stents (DES) have been proven to be effective in reducing the incidence of restenosis following percutaneous transluminal angioplasty (PTA) and by-pass surgery, many adverse effects are associated with their use. In normal vessels, the majority of vascular smooth muscle cells (VSMCs) is quiescent and possesses a contractile phenotype. In disease states or following vascular injury such as after PTA, medial VSMCs can shift from the contractile to the synthetic phenotype, re-enter the cell cycle, and migrate into the intima^5^. It is widely accepted that injury-induced pathological VSMC proliferation and migration is a major cause of neointima formation^6–8^. Therefore, finding therapies to effectively inhibit VSMC proliferation and migration is a major focus for post-intervention in cardiovascular or peripheral arterial diseases, along with endothelium protection and anti-thrombotic therapy.

The roots of *Panax notoginseng* (Burk) *F.H. Chen*, is a well-known and widely used herbal medicine in Asian countries for managing cardiovascular and cerebrovascular disease^9^. *Panax notoginseng saponins* (PNS) are the major active ingredients of Panax, with known effects in inhibiting platelet aggregation, promoting cardiac angiogenesis, improving left ventricular diastolic function, is anti-inflammatory, and inhibit vascular intimal hyperplasia and VSMC proliferation^10–13^. Notoginsenoside R1 (NGR1), a novel bisdesmosidic 20 (S) -protopanaxatriol saponin, has been identified as a unique saponin in *Panax notoginseng* but is absent in other ginseng species^14^. Mounting amount of evidence have now reported cardioprotective, neuroprotective, anti-inflammatory, and anti-oxidative benefits of NGR1^15–20^. *In vivo* and *in vitro* studies have demonstrated that NGR1 attenuates ischemia-reperfusion (I/R) injury and exhibit pro-angiogenic activity on human umbilical vein endothelial cells via VEGF-KDR/Flk-1 and phosphatidylinositol 3-kinase (PI3K)/Akt-eNOS signaling pathway^21^. In VSMC, NGR1 reduces the production of fibronectin and plasminogen activator inhibitor-1 (PAI-1) induced by TNF-α via ERK/PKB inhibition^20,22^. These findings support a protective role of NGR1 in preventing thrombosis and supporting the endothelium. However, the role of NGR1 in modulating neointima formation and VSMC function remain largely unknown. In the current study, we sought to determine the effect of NGR1 on VSMC proliferation and migration, and its ability to attenuate neointima formation *in vivo*. Our data points to a model wherein NGR1 can potently inhibit serum-induced VSMC proliferation and migration, and attenuate neointima formation in a mouse acute femoral artery injury model through Akt signalling. Our results reveal for the first time that NGR1 is a potential agent for treatment of restenosis after PTA.

## Results

### NGR1 attenuates neointima formation after femoral artery injury *in vivo*

**Figure 1:**
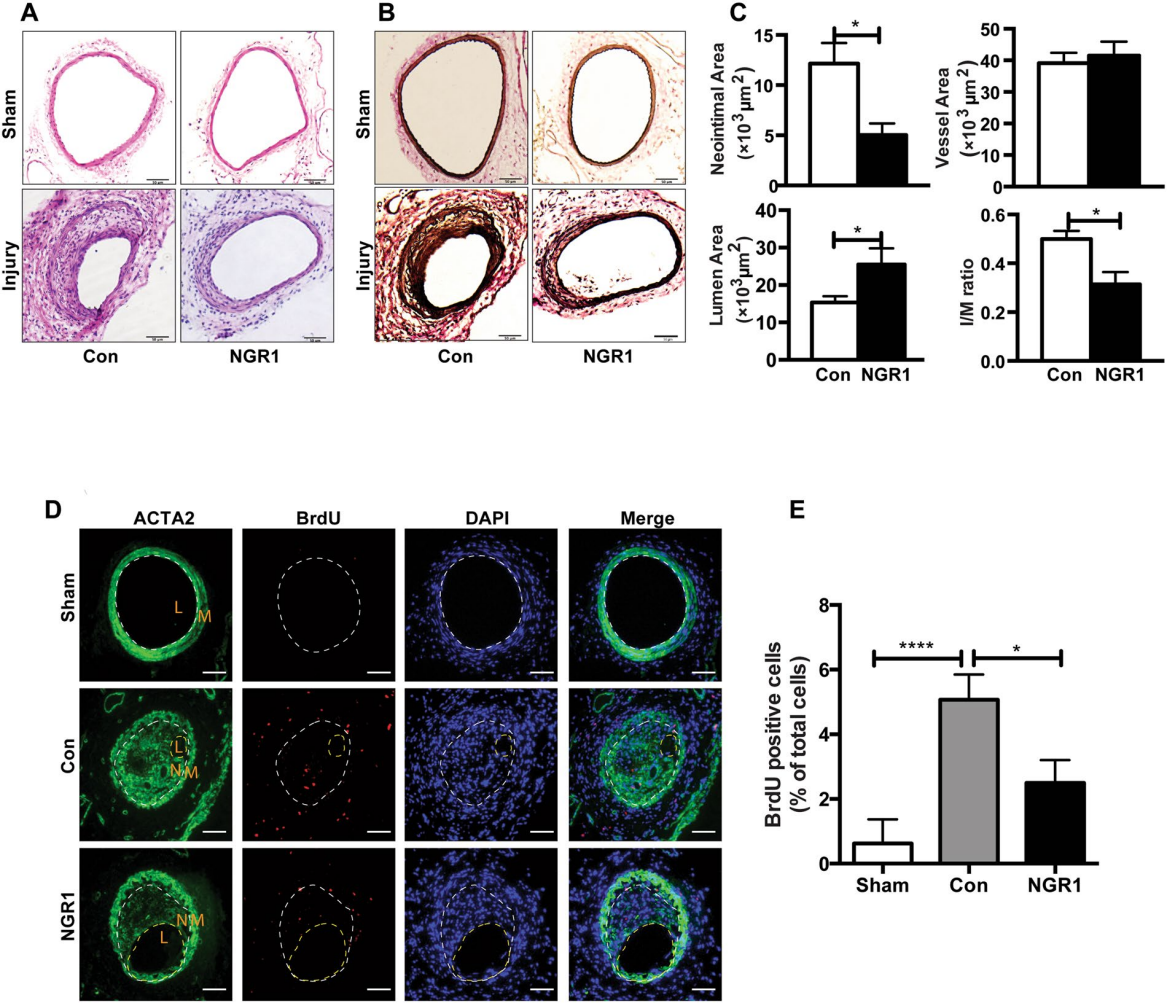
NGR1 attenuated neointima formation in a murine femoral artery wire injury model. Eight week-old C57BL/6 J male mice were intraperitoneally injected with NGR1 (10 mg/kg/d) or saline (Con) daily for three weeks. (**A**) H&E staining and (**B**) elastic van Gieson (EVG) staining of non-injured right (Sham) and injured left femoral arteries. Scale bar, 50 μm. (**C**) Morphometric analysis of neointima area, intima-to-media ratio, lumen, and vessel area of injured femoral arteries from mice injected with vehicle or NGR1. (**D**) Representative immunofluorescence stained images of the sections from sham and wire-injured femoral arteries. Scale bar, 50 μm. (**E**) Percentage of BrdU positive cell number was quantified in sections from sham operated and injured femoral arteries from vehicle or NGR1 treated mice. Data shown are means ± SEM. N = 4 for control and N = 5 for NGR1 treatment. *P < 0.05; compared with control group. Three independent experiments were performed.

To determine whether NGR1 (chemical structure showed in Supplementary Fig. 1) has an effect on neointimal hyperplasia, we performed the mouse femoral artery injury model in C57BL/6J mice injected with vehicle or NGR1. In this model, the endothelial layer of the femoral artery is denuded by passaging of a guided wire, and extensive neointima is formed as a result of medial VSMC proliferation and migration^23–25^. No differences in the gross vessel morphology were observed in sham-operated femoral arteries of mice given systemic injection of vehicle or NGR1 (Fig. 1A,B, top panels). Three weeks following femoral arterial wire injury, robust neoinitma formation was observed in vehicle treated mice. In contrast, the NGR1 treated mice displayed significantly reduced neointima hyperplasia (Fig. 1A,B, bottom panels). Morphometric analyses corroborated these histological observations where NGR1 treatment significantly attenuated neointima area, intima/media (I/M) ratio, increased lumen area, while having no effect on total vessel area (Fig. 1C). Pathological vascular remodeling following injury is attributed to increased VSMC proliferation^26^. We evaluated VSMC proliferation via 5-bromo-3′-deoxyuridine (BrdU) incorporation in smooth muscle actin (ACTA2) positive cells *in vivo*. As shown in Fig. 1D, we observed increasing numbers of BrdU labeled VSMC in the injured vehicle-treated femoral arteries (Fig. 1D, middle panel) compared to uninjured sham controls (Fig. 1D, top panel). Notably, NGR1 treatment was able to significantly reduce the number of BrdU-positive cells detected in the injured vessels (Fig. 1D, bottom panels and Fig. 1E). Together, these results suggest that NGR1 treatment reduced neointima formation by inhibiting VSMC proliferation.

### NGR1 inhibits VSMC proliferation and migration, but has no effect on cell viability and apoptosis

**Figure 2:**
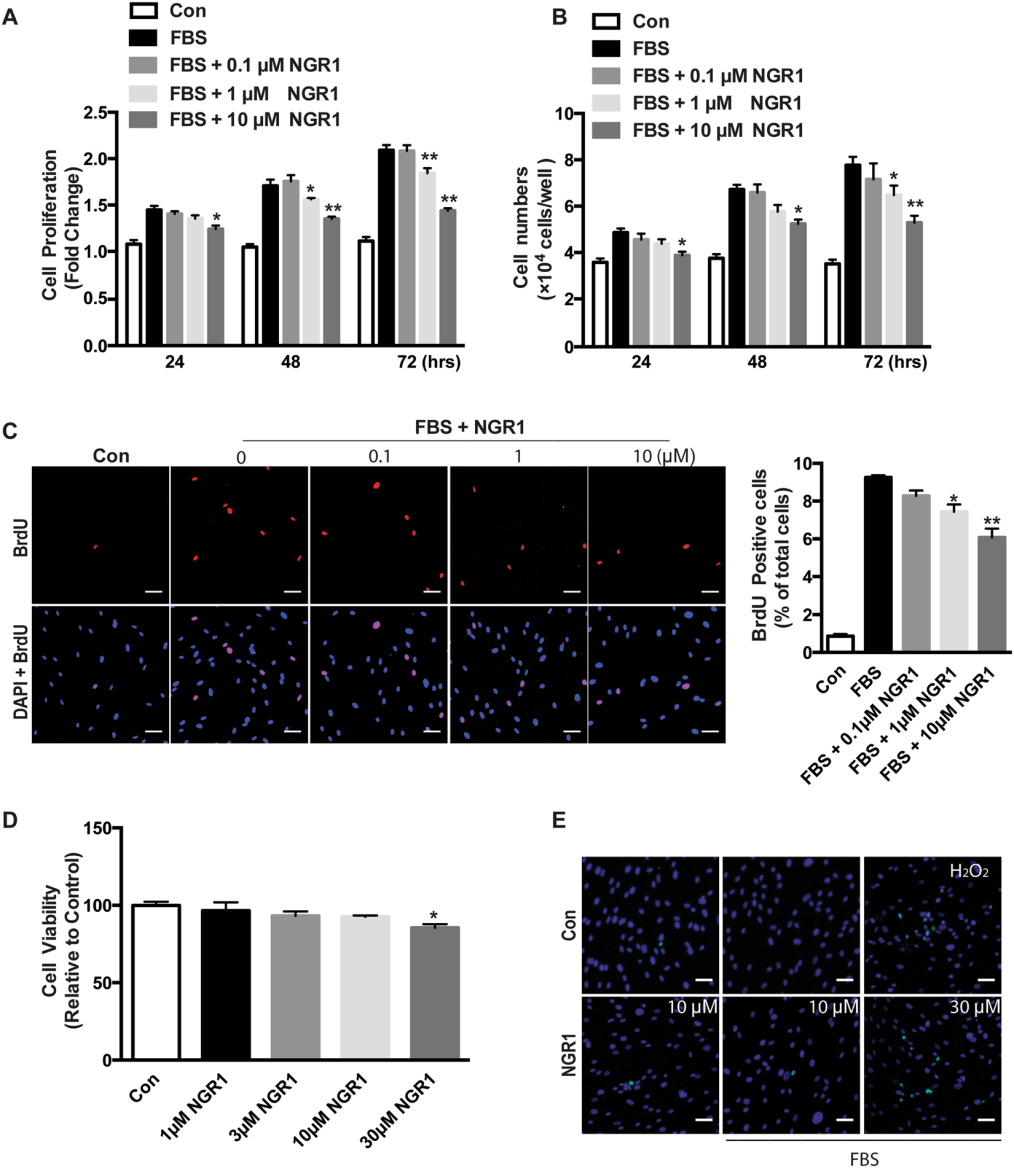
NGR1 inhibits hCASMCs proliferation but not cell viability. Serum-deprived hCASMCs were pretreated with 0.1–10 μM of NGR1 for 24 h and incubated with/without serum in the absence or presence of NGR1 for the indicated times. Cell proliferation was detected by (**A**) MTT assays, and (**B**) direct cell counting. (**C**) DNA synthesis was measured by immunofluorescent staining to detect BrdU incorporation. (**D**) Cell viability was measured by MTT assays. (**E**) hCASMC apoptosis was measured by TUNEL staining. Data shown are means ± SEM. N = 4. *P < 0.05, **P < 0.01 compared with serum control group. Scale bar, 5 μm. All experiments were repeated as least for 3 times.

Aberrant VSMC proliferation is a hallmark of restenosis and atherosclerosis formation and progression. To further investigate the possible mechanisms underlying NGR1’s ability to attenuate neointima hyperplasia, human coronary artery smooth muscle cells (hCASMC) were stimulated without and with serum in the absence or presence of varying concentrations of NGR1 to determine its effects on cell proliferation, apoptosis, and migration. hCASMC cultured in the absence of serum proliferate slowly, and NGR1 had no effect on hCASMC proliferation under these basal conditions. Proliferation was significantly increased with the addition of serum, and this response to serum was attenuated by NGR1 treatment in a dose- and time-dependent manner (Fig. 2A,B). To further validate these results, we used BrdU incorporation to examine DNA replication^27^. NGR1 treatment significantly inhibited BrdU incorporation in serum-stimulated hCASMC compared to vehicle treated controls (Fig. 2C). To exclude the possibility that the anti-proliferation effect of NGR1 on hCASMC was due to cellular cytotoxicity or apoptosis, we examined hCASMC viability and apoptosis under both basal and stimulated conditions. MTT assay showed that even at the higher concentration of NGR1 (10 μM), it did not induce cell cytotoxicity. Cell death was only observed at non-therapeutic levels of NGR1 (30 μM) (Fig. 2D). TUNEL staining corroborated these data (Fig. 2E).

**Figure 3:**
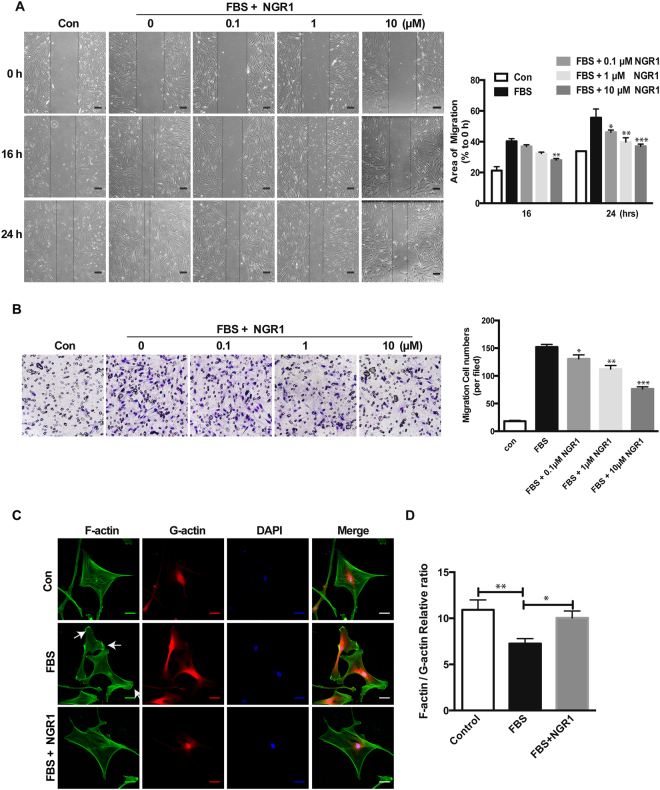
NGR1 prevents hCASMC migration through reorganization of actin cytoskeletal dynamics. (**A**) Representative images of quiescent hCASMCs that were pretreated with NGR1 for 24 h and stimulated with serum for 24 h. Images were captured at 0, 16, 24 h. Quantification of percentage of area covered by migrated cells over time was calculated using ImageJ (right panel). N = 3. Scale bar, 50 μm. (**B**) Representative images from transwell migration assay with different conditions (left panel). Quantification for the number of migrated cells are shown in the bar graph on the right. N = 3. (**C**) Representative images of F-actin (Alexa-488-tagged phalloidin), G-actin (Alexa-594-tagged DnaSe1) and DAPI stained hCASMCs that were unstimulated (Con) or treated with FBS or FBS + NGR1 (10 µM). Lamellipodia are indicated by the white arrows. Scale bar, 2 μm. (**D**) Quantitative results show the ratio of F-actin and G-actin on the basal plane of hCASMC images. Fifty cells in each condition were quantified. All experiment was repeated at least 3 times. Data shown are means ± SEM. *P < 0.05, **P < 0.01, ***P < 0.001 vs. serum control group.

In addition to VSMC proliferation, increased VSMC migration is another key process in promoting neointima formation^6–8^. To explore the potential effect of NGR1 on hCASMC migration, we performed 2D wound-healing and 3D transwell migration assays. As shown in Fig. 3A, NGR1 dose-dependently inhibited serum induced hCASMC migration and wound closure. The vehicle treated group had a 55.57 ± 9.86% scratch wound closure, while treatment with 0.1 μM, 1 μM and 10 μM of NGR1 reduced the ratio of wound closure to 46.14 ± 2.52%, 39.79 ± 4.79%, 37.02 ± 2.48%, respectively. Results from our modified Boyden chamber transwell assay further confirmed the inhibitory effect of NGR1 on hCASMCs migration where NGR1 dose-dependently inhibited the serum-induced hCASMC transmigration (Fig. 3B). These results indicate that NGR1 inhibits VSMC proliferation and migration but did not adversely affect cellular viability.

### NGR1 regulates VSMC actin organization under serum stimulation

Actin cytoskeleton in VSMC govern contractility, but also play a role in regulating VSMC motility and migration^8^. Migrating and highly mobile cells show characteristic changes in their cytoskeletal ultrastructure. Therefore, to determine whether NGR1 modulate actin reorganization as a mechanism for inhibiting hCASMC migration, we stained hCASMC with phalloidin and DNase I to detect filamentous (F-actin) and monomeric globular (G-actin) actin, respectively. In the absence of serum stimulation, F/G actin is balanced by an equilibrium reaction of actin incorporation^28^ (Fig. 3C, top panels). Exposure to serum induced rapid polarization of actin cytoskeleton and formation of lamellipodia at their leading edges which is characteristic for migrating cells (Fig. 3C, middle panels, and white arrows). NGR1 significantly reduced the number of lamellipodia in hCASMC (Fig. 3C, bottom panels). Quantification of F/G actin polymerization showed significant reduction in F/G actin ratio in hCASMC in response to serum stimulation, however this change in ratio was restored following NGR1 treatment (Fig. 3D). To determine whether NGR1 affects VSMC contractile gene expression, we examine protein expression of VSMC markers in cells with or without NGR1 treatment. We did not observe any change in Myosin-11, α-smooth muscle actin (SMA), calponin, or SM22 alpha expression under basal and serum stimulated conditions (Supp. Figure 2). These data suggest that NGR1’s inhibition of VMSC migration is through regulation of actin cytoskeleton dynamic and not contractile gene expression.

### NGR1 inhibits PI3K/Akt signaling in VSMC

**Figure 4:**
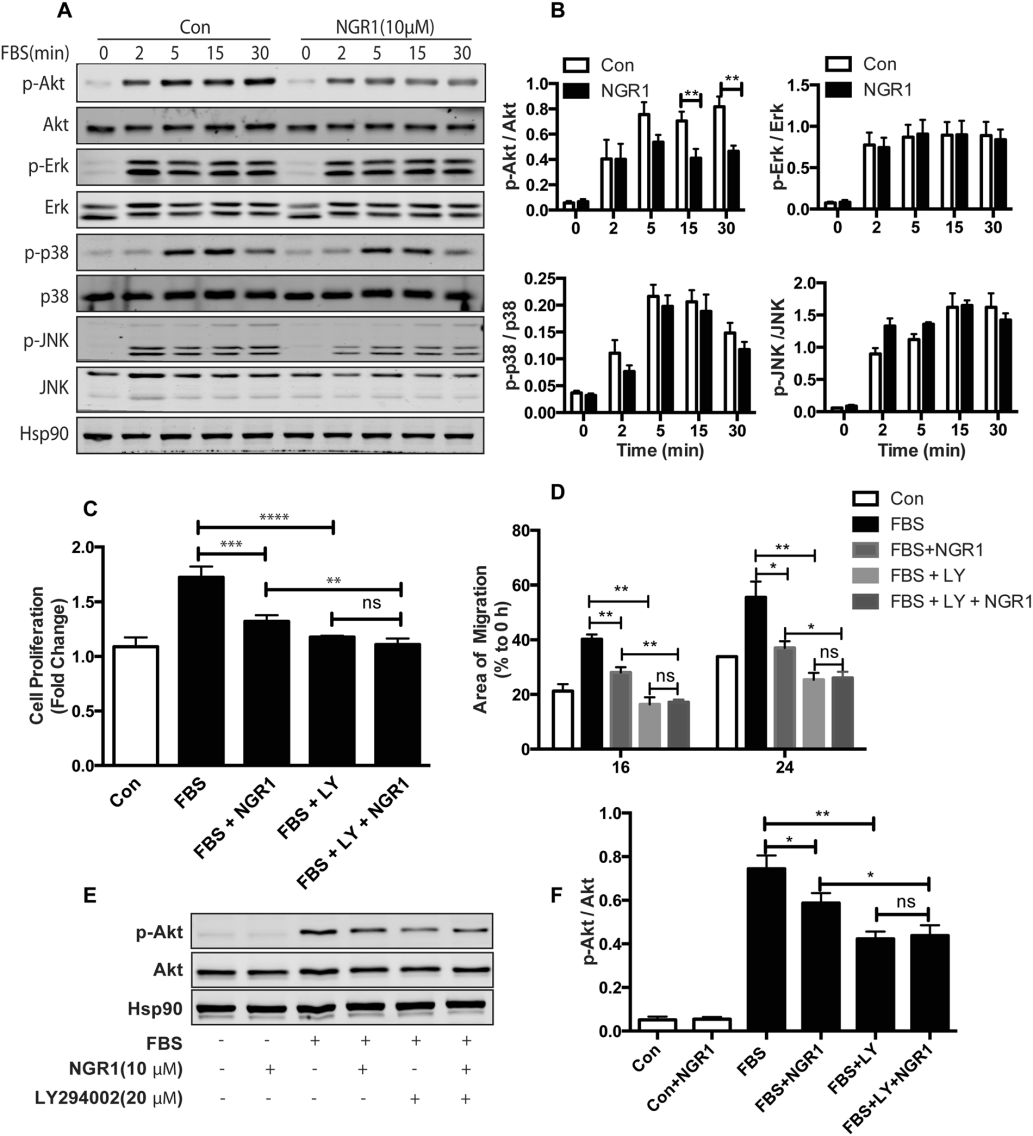
NGR1 inhibits serum-induced hCASMC proliferation and migration specifically through PI3K/Akt signaling pathway. (**A**) Western blot analysis of p-Akt/Akt, p-ERK/ERK, p-p38/p38, p-JNK/JNK in hCASMCs treated with NGR1 for 24 h and stimulated with 10% FBS for 0–30 min. Hsp90 was used as loading control. (**B**) Densitometry analysis of the phosphorylation protein normalized to total protein levels. Quiescent hCASMCs were treated with NGR1 (10 μM) for 24 h and incubated with LY294002 (20 μM) or LY294002 and NGR1 in presence or absence serum for 48 h. (**C**) Cell proliferation was quantified as fold increase of control. (**D**) Cell migration was quantified as percentage of area of migrated cells covered area relative to time 0. (**E**) Western blot of pAkt and total Akt from hCASMC treated with FBS, FBS + NGR1 with or without LY294002. (**F**) Densitometry analysis of the phosphorylation protein normalized to total protein levels of Akt under indicated condition. Data shown are means ± SEM. *P < 0.05; **P < 0.01; ***P < 0.001 compared with serum control group. All experiments were repeated for at least 3 times.

It is well known that phosphorylation and subsequent activation of PI3K/Akt and mitogen-activated protein kinase (MAPK) are the major signals involved in serum-stimulated VSMC proliferation and migration^29,30^. To determine whether NGR1 affect the PI3K/Akt or MAPK pathways, we compared levels of Akt, ERK1/2, p38, and JNK phosphorylation in hCASMCs treated with vehicle or NGR1. We observed rapid decrease in Akt phosphorylation in hCASMCs following NGR1 treatment in a time and dose dependent manner, but no effect on ERK1/2 and JNK signaling was observed (Fig. 4A,B and Supp. Figure 3). NGR1 caused a modest decrease in phosphorylation of p38 MAPK, but this did not reach significance (Fig. 4A,B).

To further validate that NGR1 acts through PI3K/Akt signaling, we tested the effect of inhibiting PI3K/Akt, ERK, p38 or JNK signaling in cultured hCASMC with their respective specific small molecule inhibitors LY294002, PD98059, SB203580, and SP600125. We compared cellular proliferation, migration and Akt activity in hCASMC at baseline, with serum stimulation in the presence of NGR1, treated with the pharmacological inhibitors, or both. hCASMC proliferation was significantly reduced in the presence of NGR1 or LY294002 compared to serum treated cells. Notably, co-treatment of cells with LY294002 and NGR1 showed that NGR1 had no effect in further reducing cell proliferation compared to LY294002 treated cells alone (Fig. 4C). On the other hand, co-treatment of NGR1 with the ERK, p38 or JNK inhibitors showed synergistic effect on VSMC proliferation (Supp. Figure 4). These data suggest NGR1 inhibits VSMC proliferation, at least in part, through PI3K/Akt but not ERK, p38 or JNK MAPK signaling pathway. The same observations were seen for migration and Akt activity (Fig. 4D,E). Furthermore, the PI3K/Akt inhibitor LY294002 decreased FBS induced lamellipodia formation in VSMC, and addition of NGR1 did not have any addictive effect on further reducing lamellipodia formation (Supp. Fig. 5). Taken together, these data indicate that NGR1 specifically target the PI3K/Akt signaling pathway in hCASMC to inhibit cell migration and proliferation, which may have contributed to the attenuation of neointima hyperplasia.

## Discussion

In the current study, we used a mouse model of injury-induced vascular neointima hyperplasia to determine whether a novel saponin, NGR1, has potential pharmacological effects on preventing pathological intima formation and vascular remodeling. NGR1 significantly attenuated neointima formation by inhibiting VSMC proliferation. Mechanistically, we demonstrated that NGR1’s ability to inhibit VSMC proliferation and migration is through inhibition of the PI3K/Akt signaling pathway and regulation of actin dynamics.

It is well know that VSMCs exist in a quiescent and differentiated state in the normal mature arterial wall, and control the vascular tone through their contractile machinery. In pathological vascular injury, VSMCs become activated and switch to a synthetic phenotype with a high rate of proliferation and migration, change in cytoskeleton composition, increased release of cytokines and chemokines, that together contribute to impaired vascular remodeling and neointimal formation^31,32^. Therefore, treatments for restenosis after PTA has largely focused on targeting VSMC proliferation and migration, and as such, drugs such as rapamycin (sirolimus) and paclitaxel (Taxol), which inhibits VSMC proliferation and migration, have been widely used in drug-eluting stents to prevent in-stent restenosis^33,34^. *Panax notoginseng* saponins (PNS) are the main active ingredient of *Pana notoginseng*, and NGR1 is one of 27 saponins identified to date. NGR1 is the characteristic ingredient of PNS in Panax and absent in other ginseng species^14^. It has been shown to have a myriad of effects including inhibiting platelet aggregation, downregulating TNF-α induced fibronectin expression, and decreasing PAI-1 production in VSMC^20,22,35^. However, the direct action of NGR1 on neointimal hyperplasia or vascular remodeling has not been investigated. Our current study found that NGR1 attenuated neointima formation caused by endothelium denudation injury while maintaining the lumen diameter (Fig. 1A–C). The therapeutic benefit of NGR1 lies in its ability to inhibit excessive VSMC proliferation as demonstrated by decreased BrdU incorporation (Fig. 1D,E, and Fig. 2A–C).

PI3K/Akt^36,37^ and MAPK^38–40^ are important signaling pathways for growth factors or cytokines (such as PDGF, interleukin-1, interleukin-6, and TNF-α) induced VSMC proliferation and migration^41^. Published data support that MAPK and PI3K/Akt signaling are differentially activated to regulate vascular remodeling and VSMC proliferation and migration. For example, Vinpocetine, an alkaloid extracted from the periwinkle plant Vinca minor, specifically reduced PDGF-stimulated VSMC proliferation and migration by inhibiting phosphorylation of ERK1/2, but not Akt^42^. In the current study, we show that NGR1 attenuated serum-induced Akt but not ERK1/2, JNK and p-38 MAPK activation (Fig. 4A,B and Supp. Figure 3). Furthermore, pharmacological inhibition of PI3K/Akt, but not those antagonizing ERK, JNK or p-38 MAPK, abolished the effect of NGR1 (Fig. 4C–F and Supp. Figure 4). These data support that NGR1 inhibition of VSMC proliferation and migration is specifically through PI3K/Akt signaling.

VSMC migration involves cell polarization, lamellipodia and filopodia formation, attachment, contraction and detachment. Actin polymerization and depolymerization play a fundamental role in these critical steps of cell migration. In mature and quiescent VSMC, G-actin polymerizes into F-actin that enable VSMC to modulate their contractility. In contrast, VSMCs extend lamellipodia toward the stimulus of pro-migratory factors via actin polymerization involving F-actin depolymerization to G-actin during VSMC migration in response to vascular injury or inflammation^8,43^. In our study, serum-stimulation induced VSMC lamellipodia formation, which was significantly reduced by NGR1 (Fig. 3C). Similarly, serum-stimulation reduced F/G actin ratio, which was increased by NGR1 treatment (Fig. 3D). These findings may represent another mechanism by which NGR1 inhibited VSMC migration. Previous studies have reported that PI3K/Akt activation is critical for actin filament remodeling to promote migration of cultured VSMCs, while disruption of Akt activity reversed the migratory effect. Our data (Supp. Fig. 5) indicated that PI3K/Akt inhibition blocked serum stimulated VSMC lamellipodia formation. Addition of NGR1 to these cells could not induce further lamellipodia inhibiton. These results suggest NGR1 inhibits VSMC actin polymerization also through PI3K/Akt signaling pathway.

Previous studies have shown that total PNS could inhibit SMC proliferation and intimal hyperplasia through an ERK dependent signaling pathway, owing to other ingredients like ginsenoside Rb1 and ginsenoside Rg1^10,44,45^. NGR1 has also been shown to inhibit the expression of ERK in other organ system and disease models, such as in pulmonary arterial^46^ and neuronal degenerative disease^18^. In the current study, our data demonstrate that NGR1 inhibits VSMC proliferation, lamellipodia formation and migration through a previously unreported PI3K/Akt pathway. The different mechanism we identified indicates that NGR1 is a unique simplex compound of PNS in Panax. Our study also suggests that the effects of NGR1 are cell and tissue specific.

There are three mammalian isoforms in Akt family, Akt1, Akt2 and Akt3, which share similarities in structure but are distinct in function^47^. Akt1 is the major isoform expressed in endothelial cells, VSMCs and macrophages, and mediate cell survive, growth and proliferation^48^. Akt2 is required for rapamycin-induced VSMC differentiation^49^, while Akt3 is mainly localized to the brain and testes^50^. In this study, we demonstrated that NGR1 inhibits VSMC proliferation and migration including actin organization, but did not have an effect on VSMC phenotype switch (Supp. Figure 2). It is plausible that the anti-proliferation and anti-migration effect of NGR1 is Akt isoform specific and this warrants further study.

In summary, we identified a novel role of NGR1 in attenuating neointimal hyperplasia in response to arterial injury *in vivo*. NGR1 inhibits VSMC proliferation and migration via PI3K/Akt signaling pathway and actin dynamics. Our findings provide the first proof of concept that NGR1 can be used as a novel therapeutic agent in the management of vascular restenosis.

## Materials and Methods

### Chemical reagents

Notoginsenoside R1 (NGR1, chemical structure C_47_H_80_O_18_, molecular weight = 933, purity >98%) was purchased from Chinese National Institute for the Control of Pharmaceutical and Biological Products. rhEGF and rhFGF were purchased from Promega (Madison, WI). The small molecule inhibitors LY294002 (PI3K/Akt inhibitor), SPD98059 (ERK inhibitor), SB203580 (p38 MAPK inhibitor), SP600125 (JNK inhibitor), BrdU were obtained from Sigma-Aldrich (St. Louis, MO). Primary antibodies against Akt/phospho-Akt, ERK/phospho-ERK, p38/ phospho-p38 and JNK/ phospho-JNK were all purchased from Cell Signaling Technology (Massachusetts, USA). Antibodies against Myosin-11, SMA, Calponin, SM22-α and BrdU were obtained from Abcam (Cambridge, UK). See Supplementary Tables 1-2 for detailed information.

### Animal and mouse femoral artery wire injury

Six-week old male C57BL/6 J mice were purchased from the Jackson Laboratory. All experiments were performed in accordance with the guidelines/regulations and were approved by the Institutional Animal Care and Use Committee (IACUC) of Temple University.

Prior to wire injury, mice were given intraperitoneal injections of NGR1 (10 mg/kg/d) or vehicle control (saline) daily for three weeks. Mice were anesthetized with an intraperitoneal injection of ketamine (100 mg/kg) and xylazine (10 mg/kg), and wire-induced left femoral artery injury was performed as previously described^23,51^. For BrdU experiments, mice were injected subcutaneously (25 mg/kg) daily for three days and intraperitoneally (30 mg/kg) 12 hours prior to sacrifice. Femoral arteries were collected at the experimental endpoint and cryopreserved for histological staining. Cryosections (5 μm) were obtained for H&E, EVG, and BrdU staining. Morphometric analyses were performed as previously described^23,25^. Briefly, ten H&E and EVG stained cross sections were used for lumen, neointimal, medial and vessel area quantification. Each section was collected 50 μm apart to cover the whole injured vessel. Values were then averaged for each animal and the mean was calculated from 4-5 animals per treatment.

### Human coronary artery smooth muscle cell culture

Primary human coronary artery smooth muscle cells (hCASMC) were purchased from Lonza (#CC-2583) and cultured in M199 supplemented with 10% FBS, rhEGF (2.7 ng/ml), rhFGF (2 ng/ml), penicillin G (100 units/ml) and streptomycin sulfate (100 μg/ml). The cells were starved with starvation media containing 0.1% FBS and 0.1% bovine serum albumin (BSA) for 24 h prior to experimental treatments. Subconfluent monolayers of fourth to seventh passage hCASMCs were used in all experiment.

### Cell proliferation and viability assay

hCASMC proliferation was measured by MTT assay and direct cell counting. MTT assays were performed as described previously with minor modifications^52^. Briefly, hCASMCs (7500 cells/well) were cultured overnight in 96-well plate in starvation media for 24 h and pretreated with varying concentrations of NGR1. Cells were then stimulated with 10% FBS in the absence or presence of different concentrations of NGR1 or/and LY294002. MTT stock solution (20 μl, 5 mg/ml in PBS) was added to wells at various time points, incubated for 4 h at 37 °C, and 200 μl DMSO were added to the media to solubilize the crystal. The optical density (OD) value was measured at 570 nm. hCASMCs incubated with starvation medium was used as controls. To obtain direct cell counts, hCASMCs (3 × 10^4^ cells/well) were cultured in 24-well and treated as described above, with the exception of MTT addition. Cells were trypsinised and counted using a hemocytometer.

### BrdU incorporation and immunofluorescent staining

hCASMC DNA synthesis was measured by a BrdU incorporation assay as described previously^53^. hCASMCs were cultured overnight on 0.1% gelatin-precoated coverslips in 12-well plate, starved and pretreated with NGR1 as described for MTT assays. Cells were stimulated with serum and varying concentrations NGR1 for 12 h, BrdU (10 μM) were added and incubated for a further 12 h. Cells were then washed with PBS and fixed with 4% paraformaldehyde for 10 min, and incubated for 20 min with 0.1% Triton-X100 to permeablize the cell membrane. Cells were then treated with 1 N HCl 10 min on ice and 2 N HCl for 10 min at room temperature. Samples were then neutralized by incubation in phosphate/citric acid buffer (pH 7.4) for 10 min at room temperature. Samples were blocked with blocking buffer (5% normal goat serum, 0.5% BSA, 0.1% triton-X100 in PBS) for 1 h. Primary anti-BrdU antibody was used at a 1:40 dilution and incubated with secondary antibody (anti-rat, 1:250) for 30 min. Nuclei were visualized with DAPI. BrdU staining on frozen mouse femoral arteries were performed similarly, with the exception of DNA denature time with 2 N HCl for 30 min at 37 °C.

### TUNEL staining for detection of cell apoptosis

hCASMCs were cultured on the pre-coated coverslips for overnight and assay conditions were as describe above for the cell proliferation assay. TUNEL staining was performed using a TUNEL and Fluor 488 Apoptosis detection assay (GeneCopdia) following the manufacture’s protocol to detect apoptosis. DAPI was used as the nuclear DNA marker.

### Boyden chamber assay

Three-dimensional cell migration was performed using the 8 μm pore size polycarbonate filters modified Boyden chamber (Costar, Cambridge, MA, USA) transwell assay. hCASMCs were plated at a density of 3 × 10^4^ cells/well cultured in starvation media in the upper chamber pre-coated with 0.1% gelatin. FBS (5%) and varying concentrations NGR1 were added into the lower chamber. Cells in starvation media or 5% FBS served as controls. Cells on both sides of the filter were fixed and stained with Diff-Quik Stain Set (Dade Behring Inc.) following 6 h of incubation. Five views from each well were captured and quantified using ImageJ (NIH).

### Scratch wound assay

Confluent cells were incubated in starvation media for 24 h and scratched with a 1-ml pipette tip^54^. Cells were then treated with 5% FBS in the absence or presence of different concentrations of NGR1 or/and LY294002 for 16 or 24 h. Images were captured at 0, 16 and 24 h with an inverted Olympus IX71microscopes and digital camera. Quantification was made by using ImageJ.

### F-actin and G-actin cytoskeleton staining

hCASMCs were seeded onto glass coverslips in M199 medium. Sub-confluent cells were starved and pretreated with NGR1, and then stimulated with 5% FBS for 16 h. Cells were fixed with 4% PFA for 10 min, permeabilized in 0.1% Triton X-100 in PBS for 5 min at RT, and stained with Alexa-488-tagged phalloidin (F-actin, Invitrogen) and Alexa-594-tagged DNaseI (G-actin, Invitrogen) for 30 min at RT. The nucleus was stained with DAPI. Images of cells were acquired using a ZEISS LSM 710 confocal microscope. Confocal images of the basal plane of phalloidin-labeled hCASMC were taken using the same gain and offset settings. Each group was quantified twenty cells and the average pixel intensity of each cell was quantified by image J.

### Western blot analysis

Western blot analysis was performed using standard protocol. Cells were harvested in RIPA buffer with protease and phosphatase inhibitors (Roche). Equal amounts of protein samples were separated by SDS-polyacrylamide gel electrophoresis (SDS-PAGE; 10-12% (w/v)) and transferred onto nitrocellulose membranes. Blots were blocked with 5% BSA and immunoblotted with primary and secondary antibodies described above.

### Statistical analysis

Quantitative results are expressed as means value ± SEM from at least three independent experiments. Two-way ANOVA was used to perform multiple comparisons (GraphPad Prism Software version 6.0). Two-tailed Student’s-test was applied to analyze differences between two groups. P values < 0.05 were considered statistically significant.

### Data availability

All data generated or analysed during this study are included in this published article (and its Supplementary Information files).

## Electronic supplementary material

Supplementary Information
