# Supplementary material for: Notoginsenoside R1 inhibits vascular smooth muscle cell proliferation, migration and neointimal hyperplasia through PI3K/Akt signaling
Source: Sci Rep. 2018 May 15;8:7595. doi: 10.1038/s41598-018-25874-y (PMC5953917; doi:10.1038/s41598-018-25874-y)
Supplement: Supplementary file 1 — Supplementary Information [file 41598_2018_25874_MOESM1_ESM.pdf]

Notoginsenoside R1 inhibits vascular smooth muscle cell proliferation, migration and neointimal hyperplasia through PI3K/Akt signaling

Haihong Fang[1,2,3], Shilin Yang[1], Yingying Luo[1,2], Cheng Zhang[2], Yi Rao[1], Renjing Liu[4,5], Yulin Feng[1,], Jun Yu[1,2,*]

[1]Center for translational Medicine, Jiangxi University of Traditional Chinese Medicine, Nanchang, Jiangxi 330002, China; [2]Department of Physiology and Center for Metabolic Disease Research, Lewis Katz School of Medicine, Temple University, Philadelphia, PA19140, USA; [3]School of Pharmacy, Jiangxi Science and Technology Normal University, Nanchang, Jiangxi 330013, China; [4]Agnes Ginges Laboratory for Diseases of the Aorta, Centenary Institute, University of Sydney, Sydney, Australia; [5]Sydney Medical School, University of Sydney, Sydney, Australia

Running title: NGR1 reduces neointimal hyperplasia

***Corresponding author:**

Jun Yu, MD
Department of Physiology and Center for Metabolic Disease Research, Lewis Katz School of Medicine, Temple University, Philadelphia, PA19140, USA.
Tel: 215-707-3286; Fax: 215-707-5737; Email: jun.yu@temple.edu

Supplement Figure 1

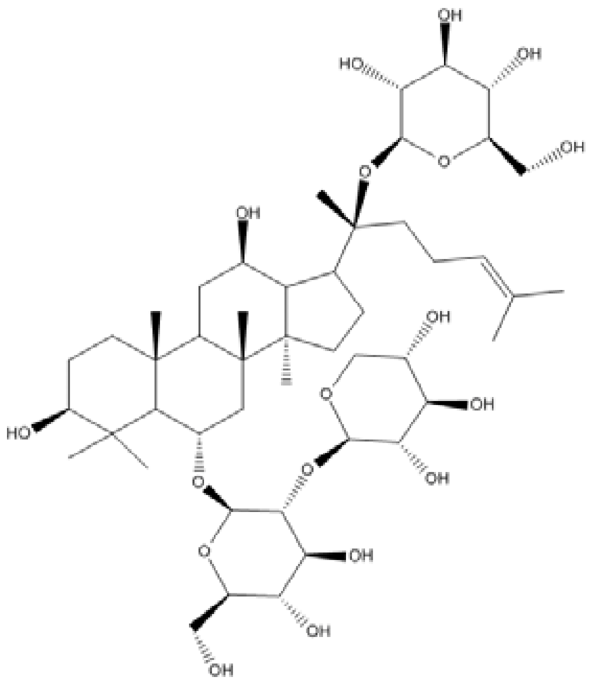

Supplement Figure 1. The chemical structure of NGR1.

Supplement Figure 2

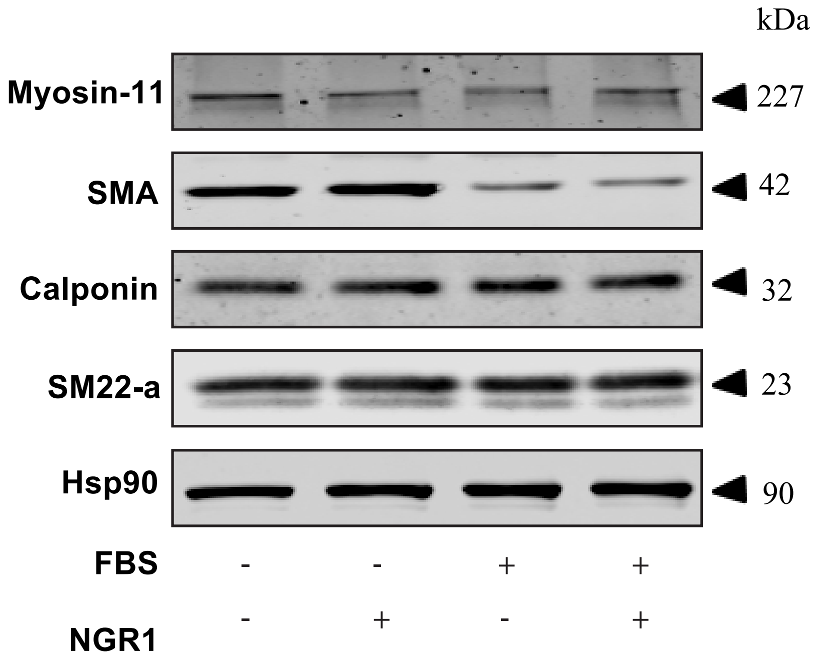

Supplement Figure 2. NGR1 treatment does not change VSMC contractile protein expression. Western blot analysis of cell lysate of hCASMCs that were treated with NGR1 for 24hrs and then stimulated with or without serum for 24hrs. The protein expression levels of Myosin-11, SMA, Calponin and SM22 alpha were blotted. Hsp90 was used as loading control. Representative western blots from 3 experiments are shown.

Supplement Figure 3

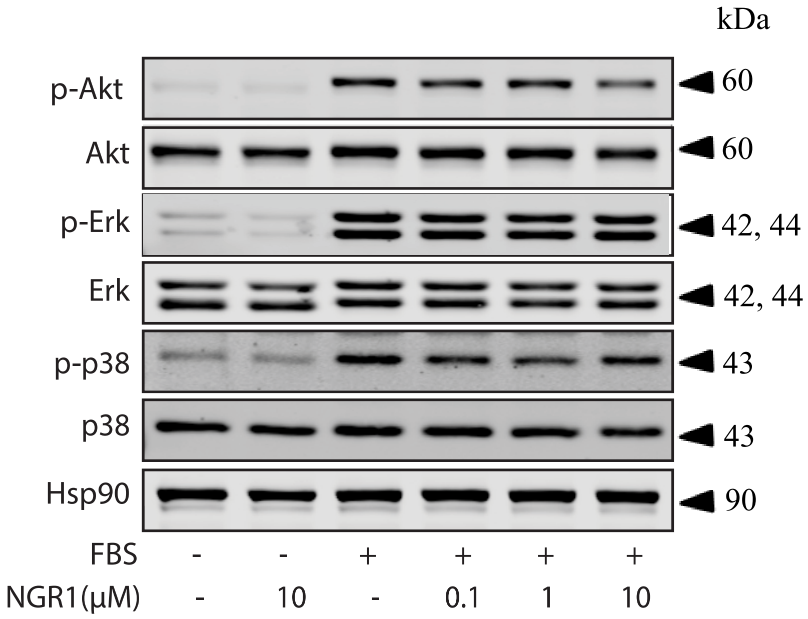

Supplement Figure 3. NGR1 inhibits serum-induced hCASMC proliferation and migration specifically through PI3K/Akt signaling pathway in dose-dependent manor.
Western blot analysis of hCASMCs that were treated with different concentration of NGR1 (0, 0.1, 1, 10 μM) for 24hrs and stimulated with 10 % FBS for 15 min. The protein expression levels of p-Akt/Akt, p-ERK/ERK, p-p38/p38 were blotted. Hsp90 was used as loading control. Representative western blots from 3 experiments are shown.

Supplement Figure 4

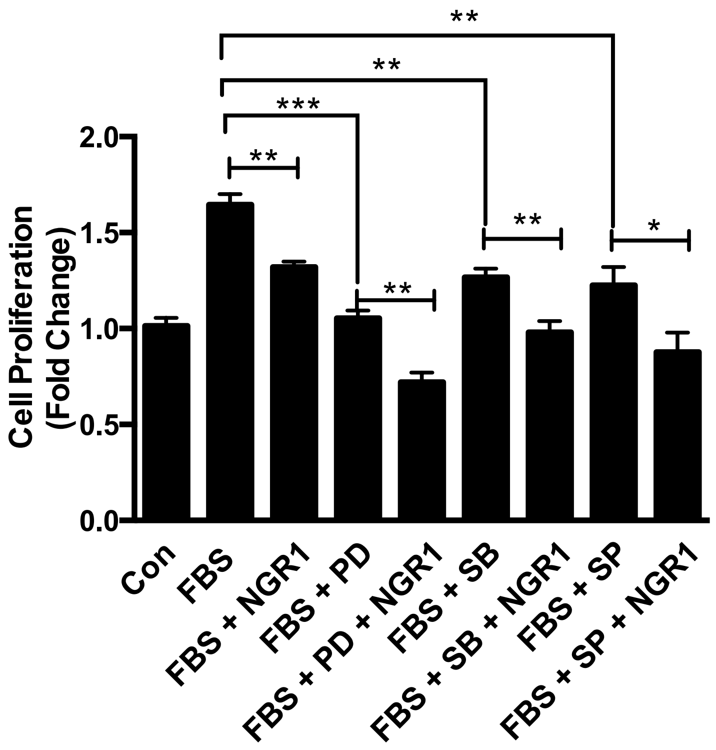

Supplement Figure 4. MAPK small molecule inhibitors doses not constraint NGR1's inhibitory effect on hCASMC proliferation.

Quiescent hCASMCs were treated with NGR1 (10 μM) for 24hrs and incubated with PD98059 (50 μM), SB203580 (20 μM) or SP600125 (20 μM) in combination of NGR1 (10 μM) or vehicle 48hrs. Cells proliferation was determined by MTT assay. N=3 for each condition. Data shown are means ± SEM. *$P < 0.05$; **$P < 0.01$; *** $P < 0.001$.

Supplement Figure 5

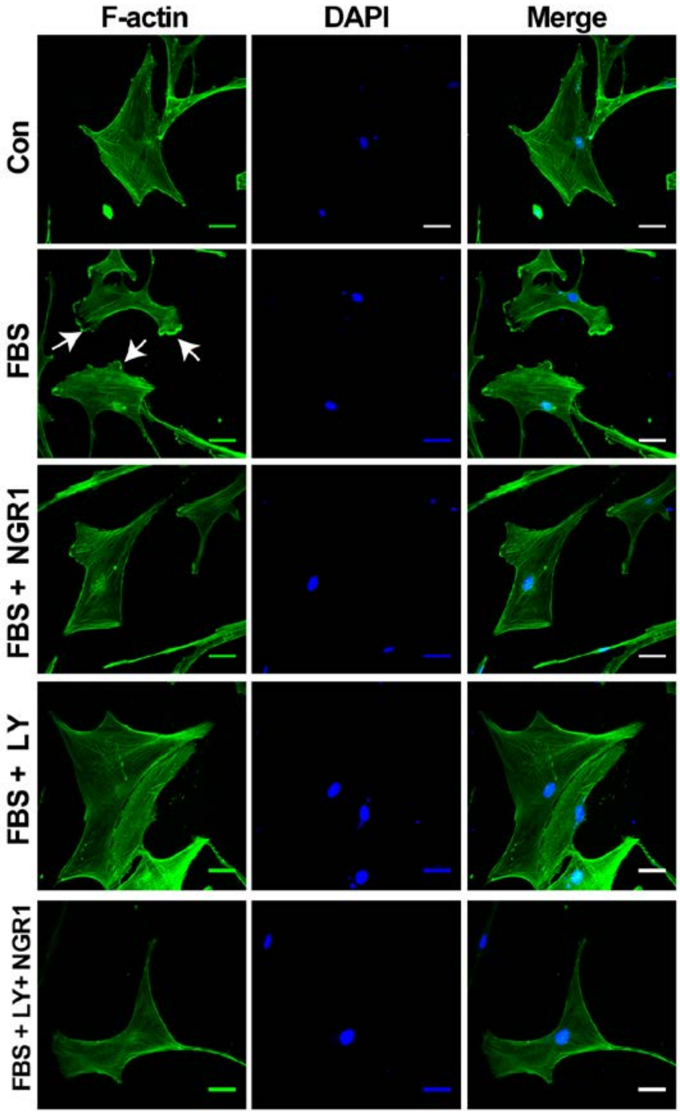

Supplement Figure 5. Representative images of F-actin (Alexa-488-tagged phalloidin) and DAPI stained hCASMCs that were non-treated (Con) or treated with FBS, FBS + NGR1 (10 µM), FBS + LY294002 (20 µM), or FBS + LY294002 + NGR1. Lamellipodia are indicated by the white arrows. Scale bar, 2 µm.

Original western blotting images for Fig. 4A.

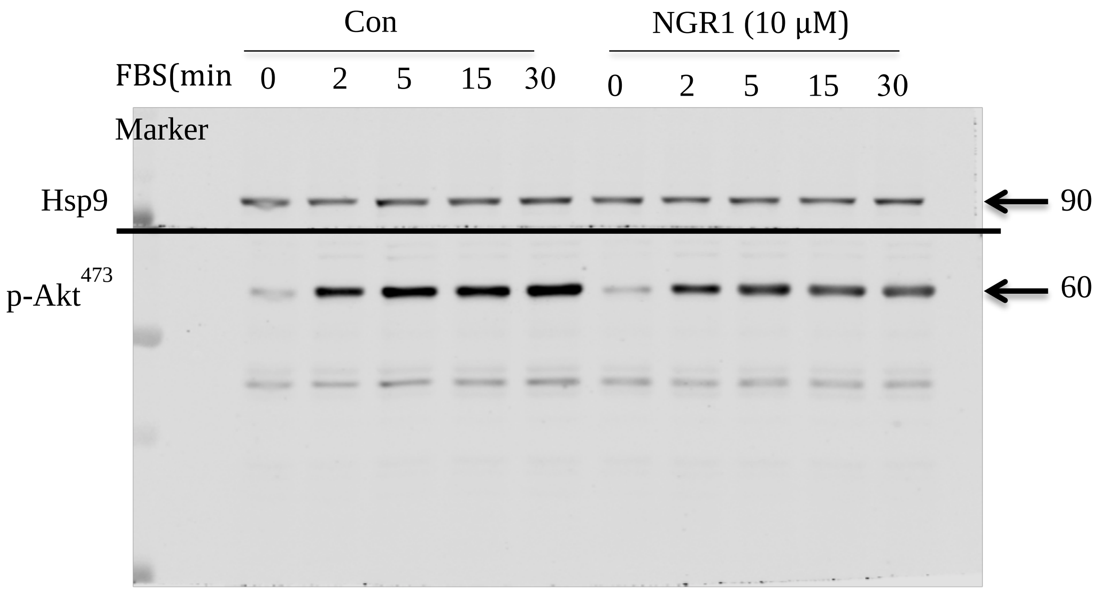

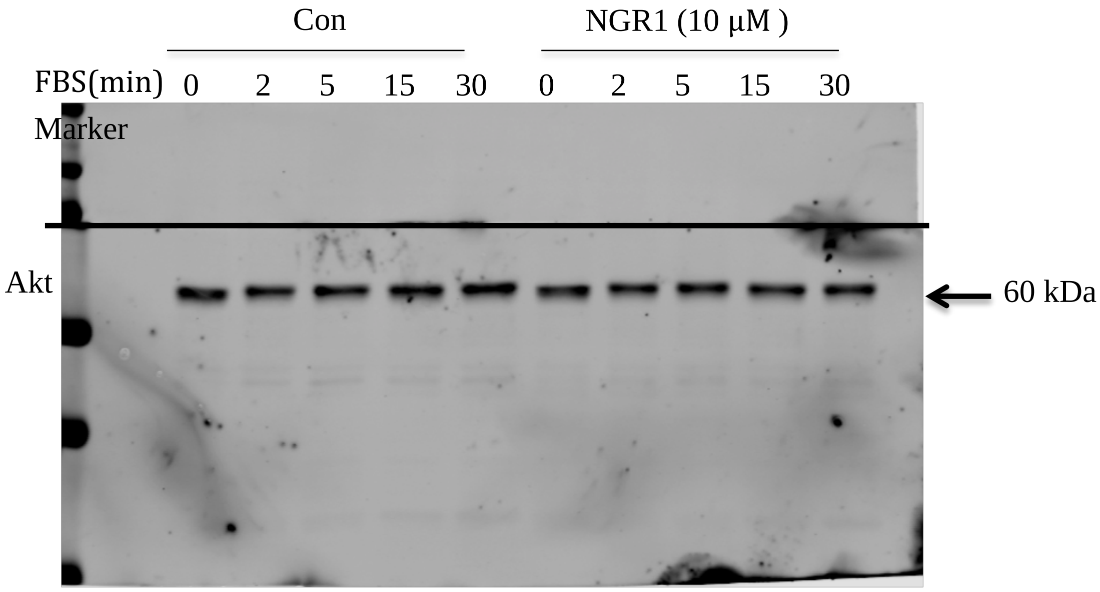

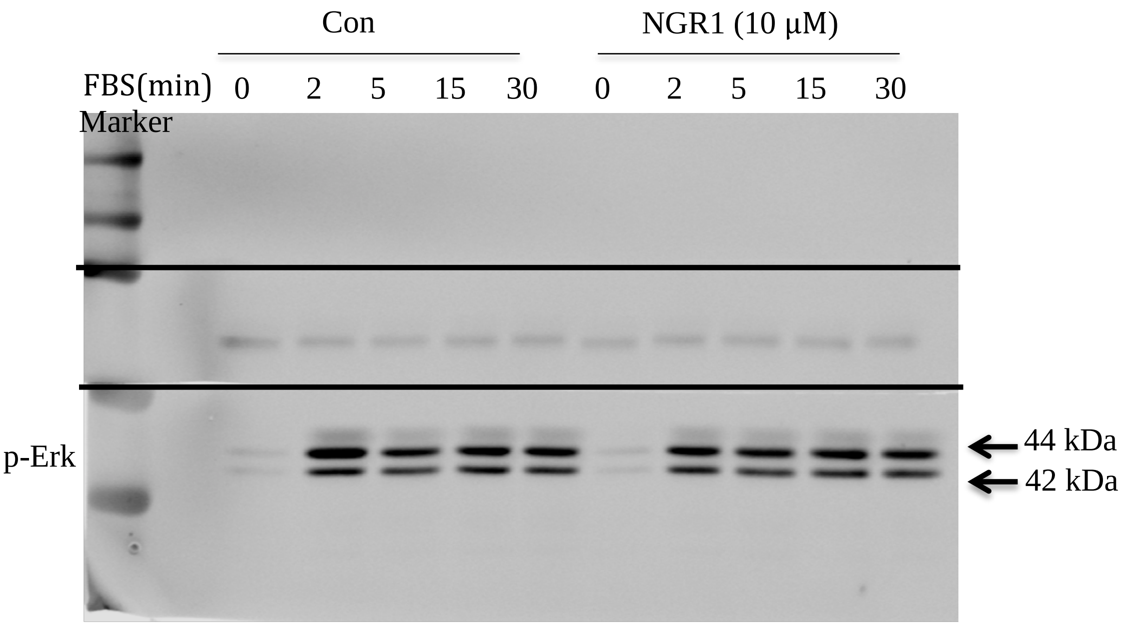

Con
NGR1 (10 μM)
FBS(min) 0 2 5 15 30 0 2 5 15 30
Marker
p-Erk
44 kDa
42 kDa

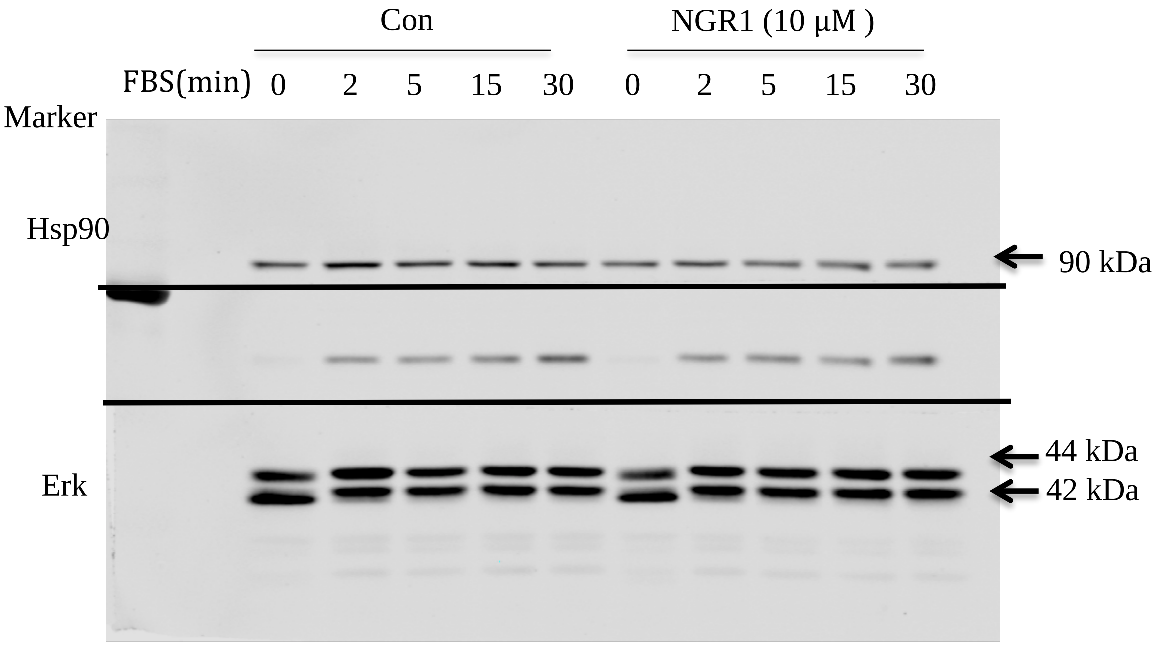

Con
NGR1 (10 μM)
FBS(min) 0 2 5 15 30 0 2 5 15 30
Marker
Hsp90
90 kDa
Erk
44 kDa
42 kDa

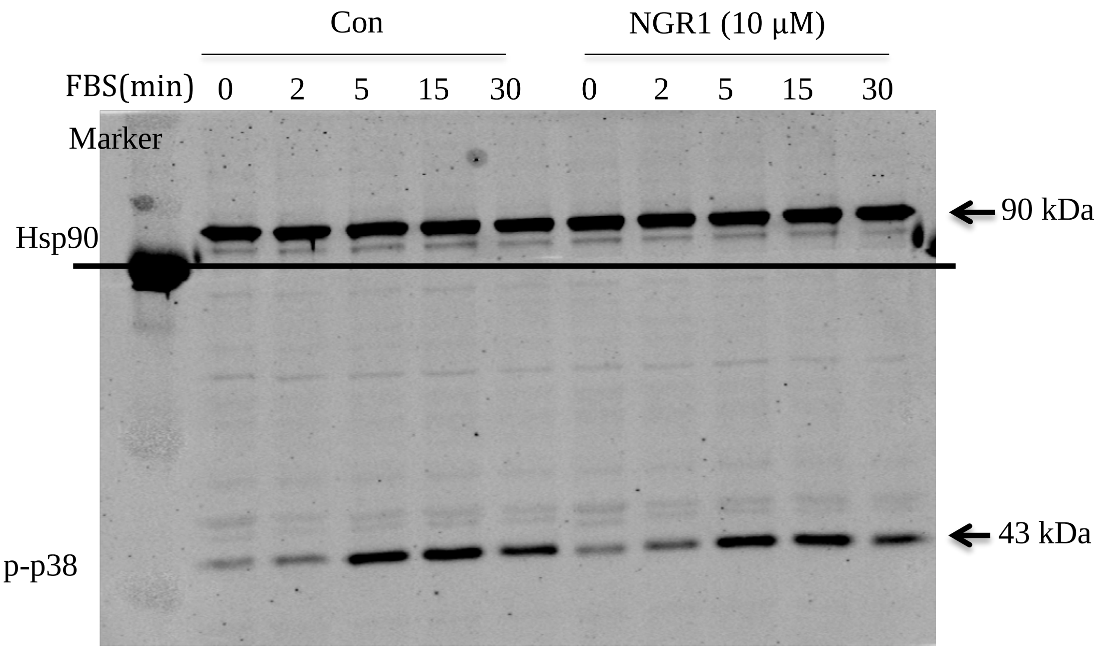
Con
NGR1 (10 μM)
FBS(min) 0 2 5 15 30 0 2 5 15 30
Marker
Hsp90
p-p38
90 kDa
43 kDa

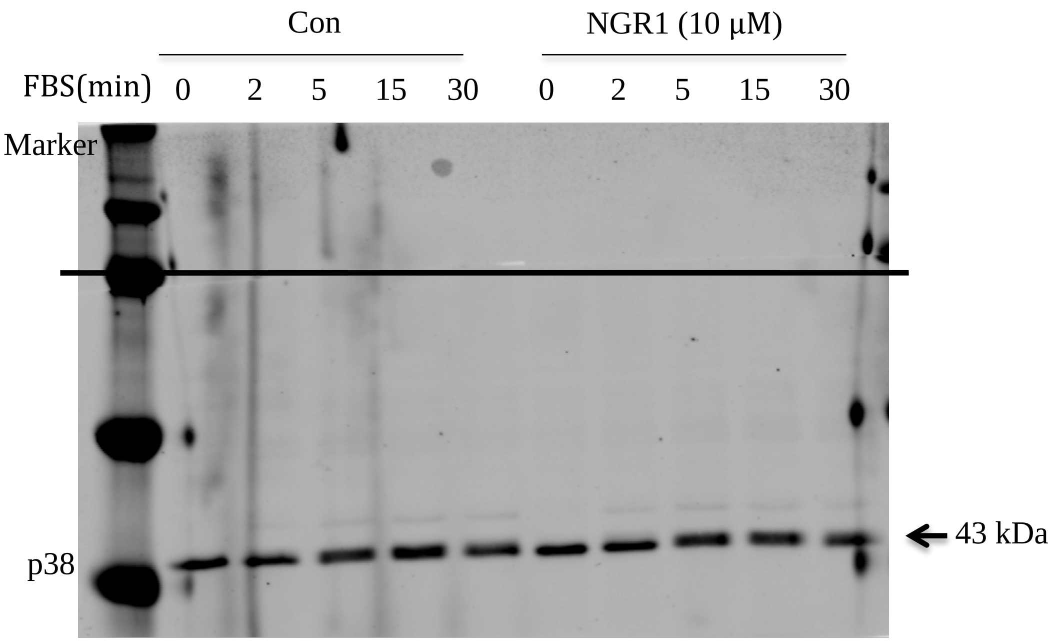
Con
NGR1 (10 μM)
FBS(min) 0 2 5 15 30 0 2 5 15 30
Marker
p38
43 kDa

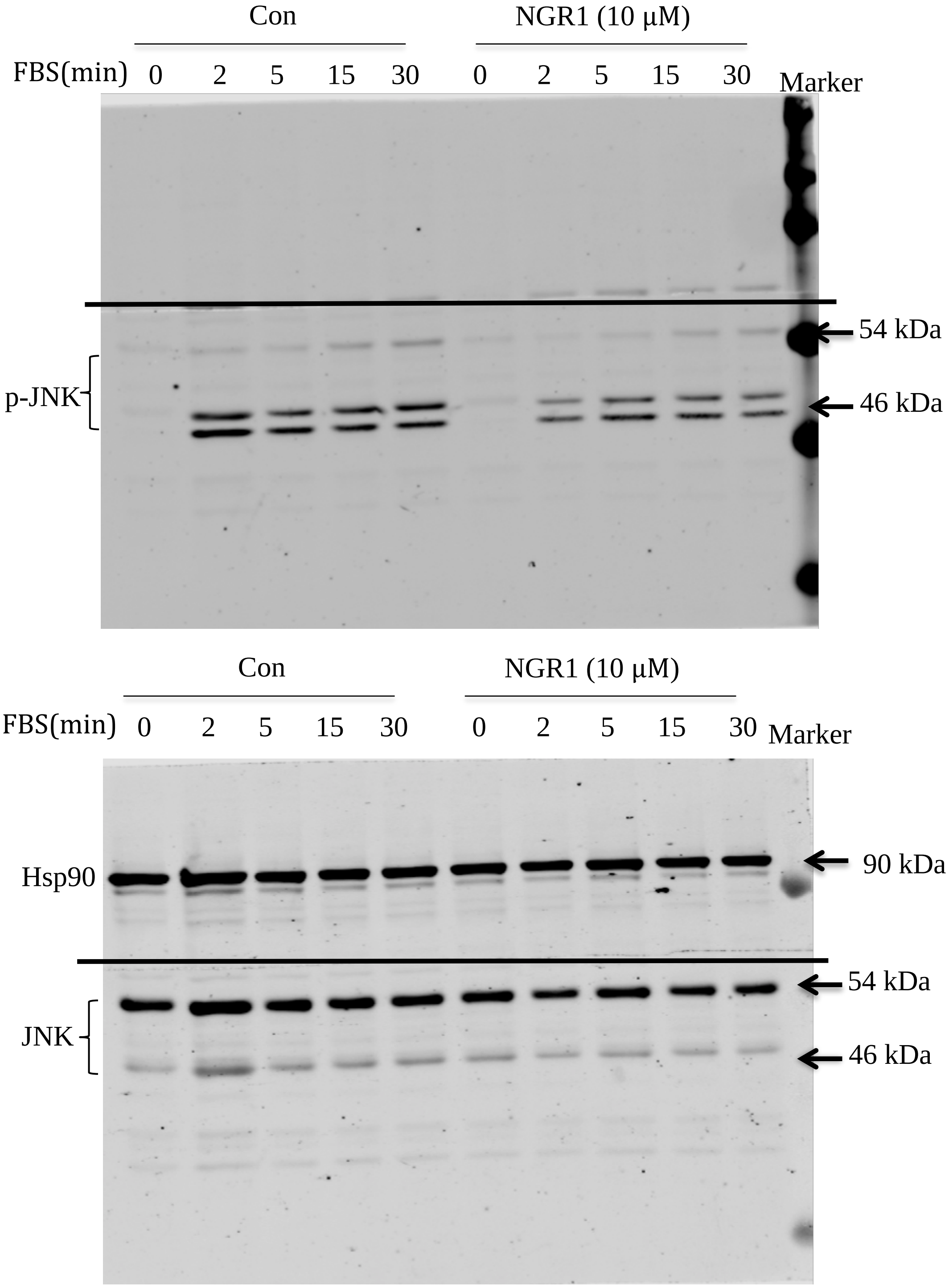
Con
NGR1 (10 μM)
FBS(min) 0 2 5 15 30 0 2 5 15 30 Marker
p-JNK
54 kDa
46 kDa
Con
NGR1 (10 μM)
FBS(min) 0 2 5 15 30 0 2 5 15 30 Marker
Hsp90
90 kDa
JNK
54 kDa
46 kDa

Original western blotting images for Fig. 4E.

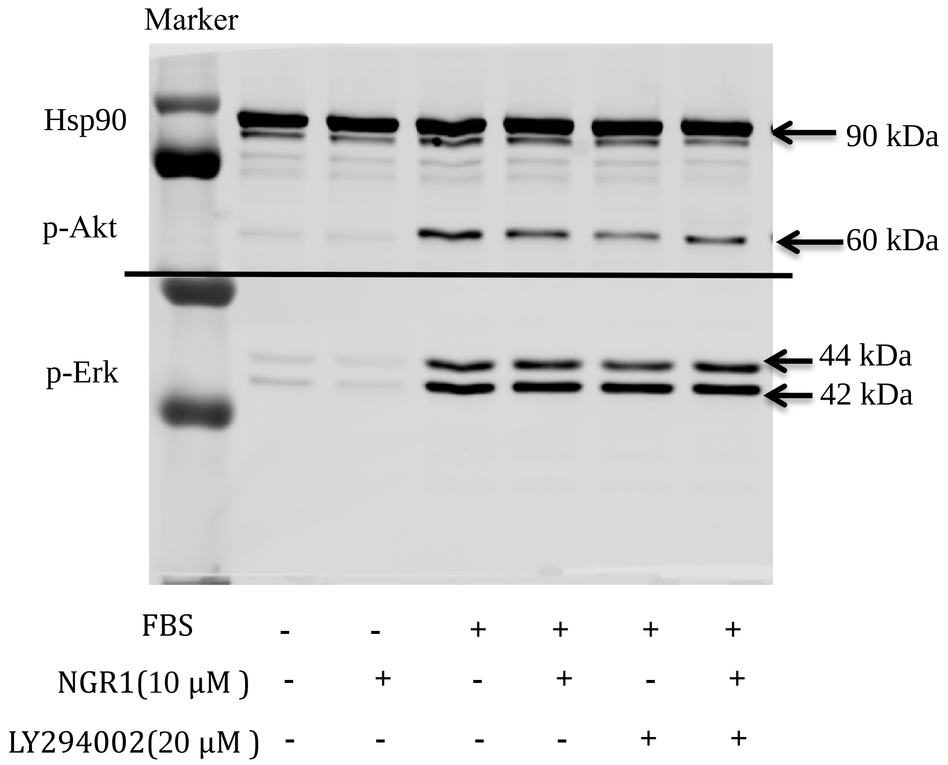

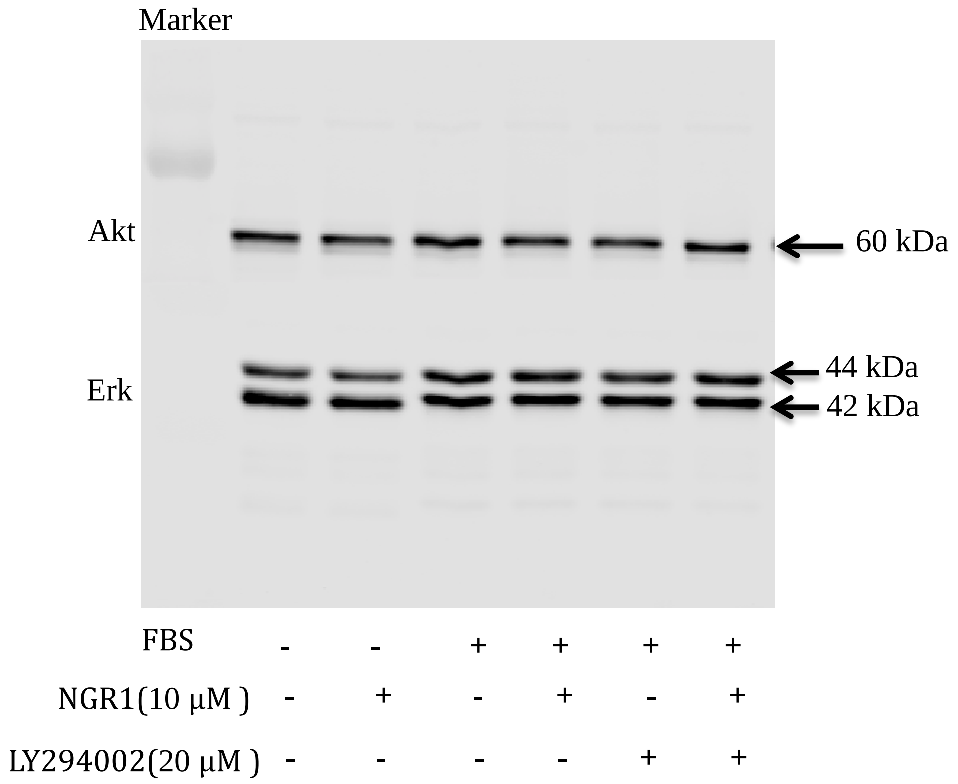

Original western blotting images for supplementary Fig. 2.

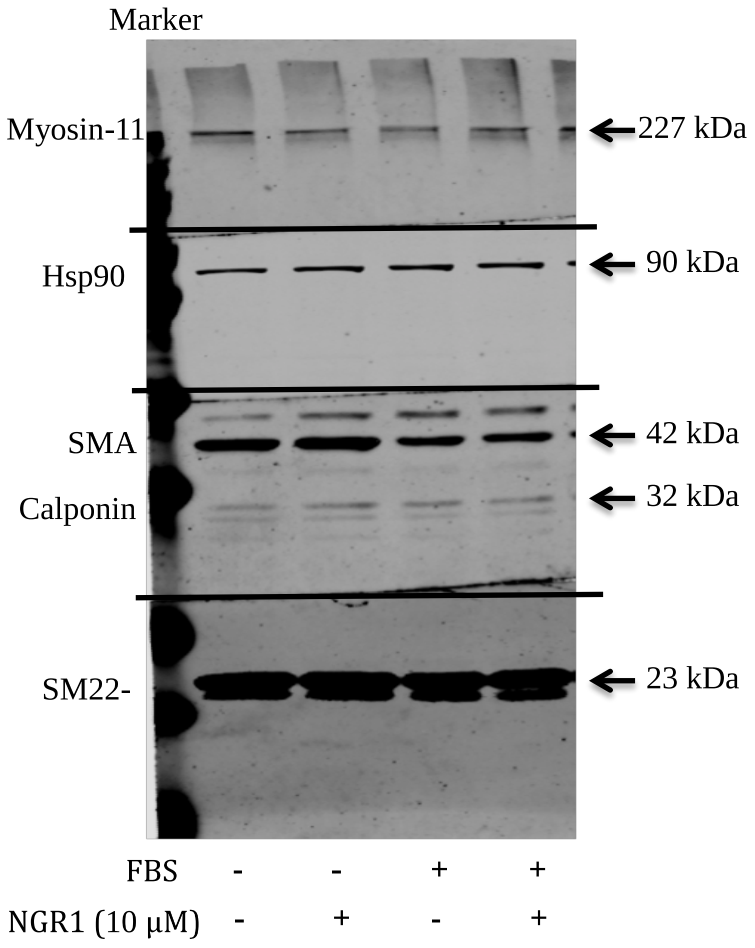

Original western blotting images for supplementary Fig. 3.

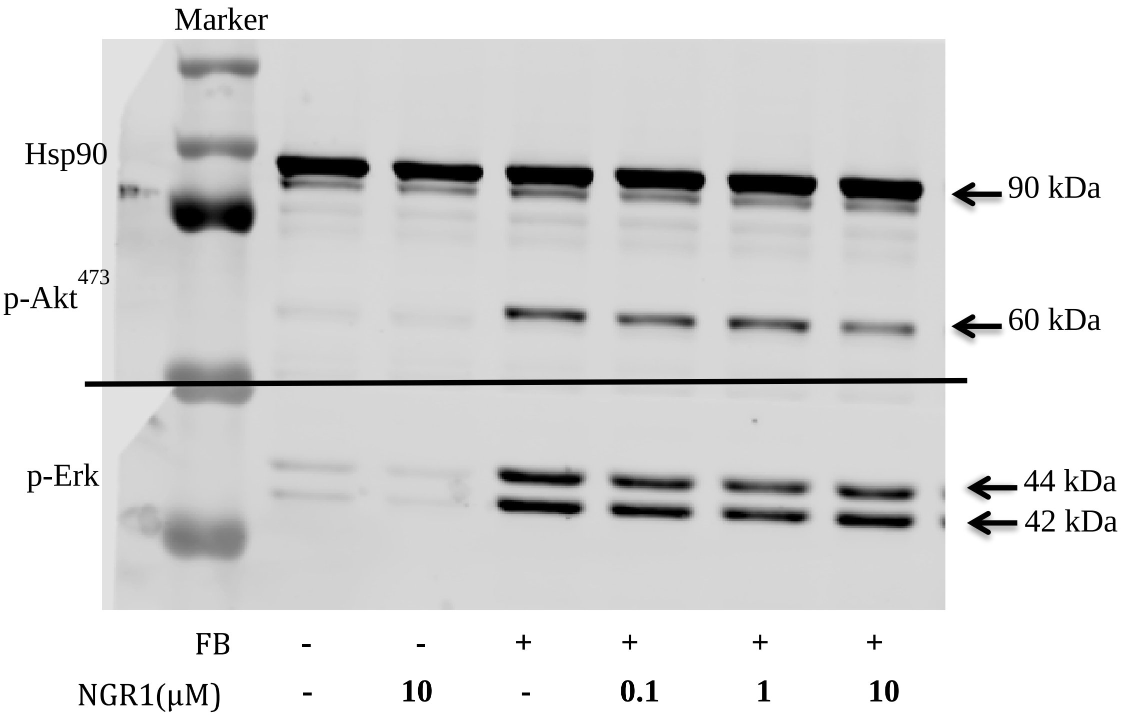

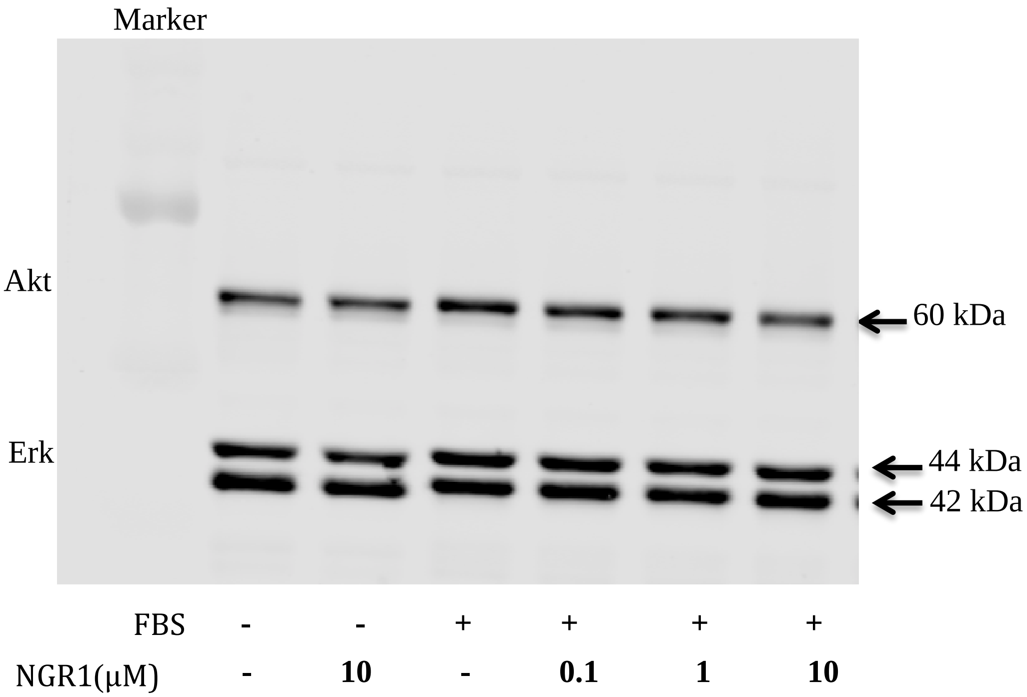

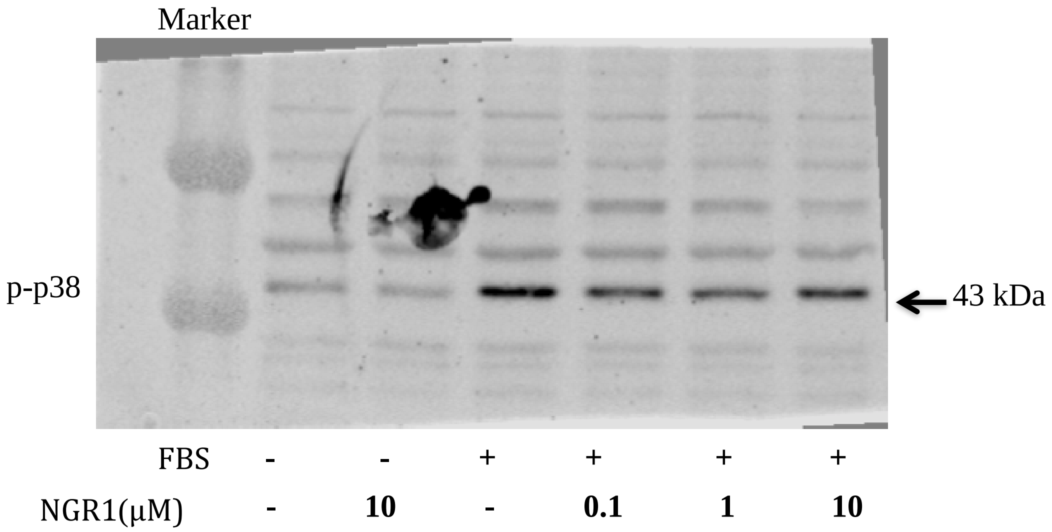
Marker
p-p38
43 kDa
FBS - - + + + +
NGR1(μM) - 10 - 0.1 1 10

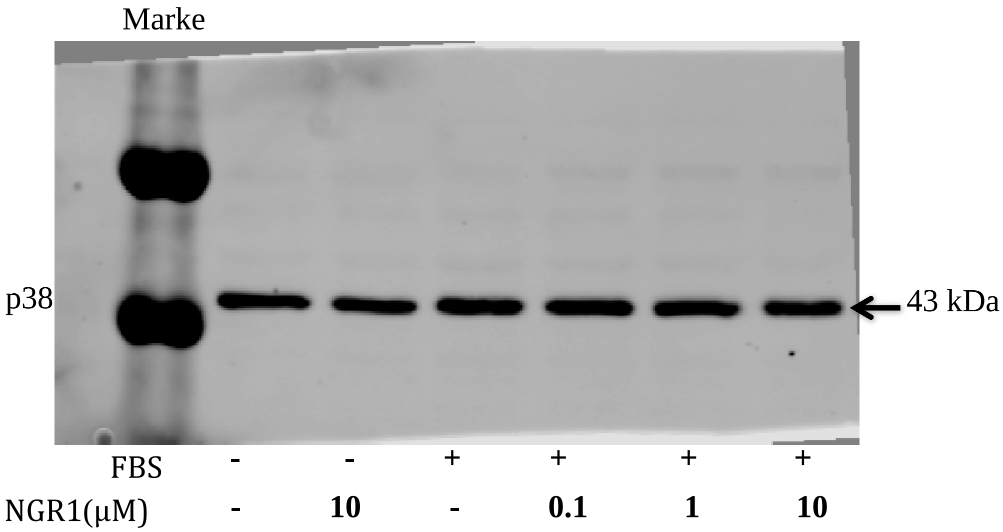
Marke
p38
43 kDa
FBS - - + + + +
NGR1(μM) - 10 - 0.1 1 10

Supplement Table 1: Antibody list

Antibody	Company	Catalog #	Species	Dilution	Application
Hsp90	BD	610419	Mouse	1:1000	Western
p-Akt(Ser473)	Cell Signaling	4051	Mouse	1:1000	Western
Akt	Cell Signaling	4685	Rabbit	1:1000	Western
p-Erk	Cell Signaling	4377	Rabbit	1:1000	Western
Erk	Cell Signaling	4696	Mouse	1:1000	Western
p-p38	Cell Signaling	9216	Mouse	1:500	Western
p38	Cell Signaling	9212	Rabbit	1:500	Western
p-JNK	Cell Signaling	4668	Rabbit	1:1000	Western
JNK	Santa Cruz	sc-7345	Mouse	1:500	Western
Myosin-11	Abcam	ab53219	Rabbit	1:1000	Western
Calponin	Abcam	ab46794	Rabbit	1:2000	Western
SM22-α	Abcam	ab14106	Rabbit	1:1000	Western
SM-α-actin	DAKO	M0851	Mouse	1:1000	Western
IRDYE 680RD second antibody	Odyssey	926-68073	Rabbit	1:5000	Western
IRDYE 800CW second antibody	Odyssey	926-32212	Mouse	1:5000	Western
Anti-BrdU	Abcam	ab6326	Rat	1:40	IF
FITC- conjugated phalloidin （F-actin）	Invitrogen	A12379		1:100	IF
Alexa Fluor® 594 conjugate Deoxyribonuclease I (G-actin)	Invitrogen	D12372		1:200	IF

Supplement Table 2: Other reagent list

Reagents	Company	Catalog #
Notoginsenoside R1	Chinese National Institute for the Control of Pharmaceutical and Biological Products	110745
rhEGF	Promega	G5021
rhFGF	Promega	G5071
BrdU	Sigma-Aldrich	19160
LY294002	Sigma-Aldrich	L9908
PD98059	Sigma-Aldrich	P215
SB203580	Sigma-Aldrich	S8307
SP600125	Sigma-Aldrich	S5567
TUNEL Andy Fluor™ 488 Apoptosis Detection Kit	GeneCopdia	A050
